# The Purple Leaf (*pl6*) Mutation Regulates Leaf Color by Altering the Anthocyanin and Chlorophyll Contents in Rice

**DOI:** 10.3390/plants9111477

**Published:** 2020-11-03

**Authors:** Asadullah Khan, Sanaullah Jalil, Huan Cao, Yohannes Tsago, Mustapha Sunusi, Ziyan Chen, Chunhai Shi, Xiaoli Jin

**Affiliations:** The Key Laboratory for Crop Germplasm Resource of Zhejiang Province, and Department of Agronomy, College of Agriculture and Biotechnology, Zhejiang University, Hangzhou 310058, China; asadkhanshervani@zju.edu.cn (A.K.); 11816104@zju.edu.cn (S.J.); 21816122@zju.edu.cn (H.C.); 11616089@zju.edu.cn (Y.T.); 11616090@zju.edu.cn (M.S.); 21716122@zju.edu.cn (Z.C.); chhshi@zju.edu.cn (C.S.)

**Keywords:** rice (*Oryza sativa* L.), *pl6*, *OsPL6*, purple leaf, anthocyanin, photosynthesis, chloroplast, hormones

## Abstract

The anthocyanin biosynthesis attracts strong interest due to the potential antioxidant value and as an important morphological marker. However, the underlying mechanism of anthocyanin accumulation in plant tissues is not clearly understood. Here, a rice mutant with a purple color in the leaf blade, named *pl6*, was developed from wild type (WT), Zhenong 41, with gamma ray treatment. By map-based cloning, the *OsPL6* gene was located on the short arm of chromosome 6. The multiple mutations, such as single nucleotide polymorphism (SNP) at −702, −598, −450, an insertion at −119 in the promoter, three SNPs and one 6-bp deletion in the 5′-UTR region, were identified, which could upregulate the expression of *OsPL6* to accumulate anthocyanin. Subsequently, the transcript level of structural genes in the anthocyanin biosynthesis pathway, including *OsCHS, OsPAL, OsF3H* and *OsF3′H*, was elevated significantly. Histological analysis revealed that the light attenuation feature of anthocyanin has degraded the grana and stroma thylakoids, which resulted in poor photosynthetic efficiency of purple leaves. Despite this, the photoabatement and antioxidative activity of anthocyanin have better equipped the *pl6* mutant to minimize the oxidative damage. Moreover, the contents of abscisic acid (ABA) and cytokanin (CK) were elevated along with anthocyanin accumulation in the *pl6* mutant. In conclusion, our results demonstrate that activation of *OsPL6* could be responsible for the purple coloration in leaves by accumulating excessive anthocyanin and further reveal that anthocyanin acts as a strong antioxidant to scavenge reactive oxygen species (ROS) and thus play an important role in tissue maintenance.

## 1. Introduction

Plants are decorated with a vast range of color through the accumulation of two major classes of pigments including flavonoids and carotenoids across the flowers, fruit, foliage, seeds and roots, etc. Flavonoids are divided into six major subgroups due to their structural complexity, including flavones, flavandiols, flavonols, chalcones, anthocyanins and pro-anthocyanidins [1]. Anthocyanin in particular accumulated in vegetative and reproductive organs, and was responsible for the orange to blue range of colors [2]. The anthocyanin accumulation in the edible part of the plant has not only offered antioxidative and anticancer properties for human consumption [3] but also played an important role in biotic and abiotic tolerance in plants [4]. Usually, rice is a green plant; however, the biosynthesis of anthocyanin imparted purple, red and black colors in different tissues, such as leaf blade, leaf sheath, stigma, pericarp, apiculus, palea, sterile lemma, awn, internode and basal leaf [5]. The purple coloration to the non-edible part was used in breeding programs as a morphological marker to identify the varieties and to study the linkage analysis [6]. Particularly, the purple apiculus color is widely used in the breeding program [7].

In the past two decades, anthocyanin pigmentation has been studied extensively. Three kinds of genes have been reported to be the basic genes to synthesize anthocyanin and named the mechanism as CAP control system. In this system, the C (chromogen) gene is the basic gene responsible for production of chromogen, activated by A (activator) and distributed by P (distributor) in specific tissues [8]. Anthocyanin biosynthesis in arabidopsis and maize was activated in seed coat and other vegetative tissues by the MBW complex, consisting of the genes of MYB, bHLH and WDR families [9]. Regulation of anthocyanin biosynthesis through the MBW system was well described in arabidopsis and maize, but more attention should be given to rice. Based on sequence similarity, initially five known orthologus genes in maize were comparatively mapped in rice including five bHLH genes and one R2R3 gene. Five homologues of the maize R and B genes have been isolated from rice and were known as Ra1/OsB1, Rb, Rc and OsB2, which were encoded by bHLH containing motif proteins [8,10,11]. The transcription factor MYB family proteins were distributed into three groups known as: R1R2R3-MYB, R2R3-MYB and R1-MYB [12]. Among them, the R2R3 type was studied most in plants. The first R2R3-MYB gene identified in *Zea mays* was *C1* [13], which was later mapped in rice for apiculus purple coloration on based of sequence similarity [14]. Furthermore, *OsC1* was studied in rice for purple coloration in apiculus and leaf sheath using residual heterozygous lines (RHLs) and somaclonal mutants, respectively [15,16]. Moreover, *OsC1* was cloned for purple coloration in stigma, apiculus and leaf sheath of rice in same year using natural variants through map-based cloning [7,17]. However, *OsC1* is not reported in purple leaves. To date, the genes involved in purple leaf of rice are not studied well and requisite to explore the mechanism behind it.

In this study, we developed *pl6* mutant from an M_2_ population of indica rice using gamma rays. This mutant had phenotypically distinct purple leaf color from tillering stage to early heading stage. The objective of this study was (1) to map the candidate gene using the map-based cloning strategy; (2) to evaluate the influence of anthocyanin accumulation on growth and physio-biochemical responses in the *pl6* mutant; (3) to identify the expression profile of associated genes involved in the anthocyanin accumulation and photosynthetic efficiency.

## 2. Material and Methods

### 2.1. Plant Material

The rice (*Orzya sativa* L.) *pl6* mutant with purple coloration in the leaf blade was developed from Zhenong 41 (*Orzya sativa* subsp. *indica*) by gamma ray irradiation. After mutagenesis, mutant was self-crossed for 10 generations until it become stable. The mutant was further crossed with its wild type (WT) and japonica cultivar, Zhenongda 104 to develop F_2_ populations for genetic analysis and gene mapping, respectively. The study was conducted in the paddy rice field of Zhejiang University, Hangzhou, China (30°15′49″ N, 120°7′15″ E) in the normal growing season.

### 2.2. Genetic Analysis and Map-Based Cloning

Plants from the F_2_ population developed by the crossing between *pl6* and its WT, grown for genetic analysis, were visually counted. Their segregation ratio of the individuals with purple leaves and green leaves were analyzed in Microsoft Excel. The F_2_ population constructed by the crossing between *pl6* and Zhenongda 104 (*Oryza sativa* L. ssp. *japonica*) were developed for the gene mapping. Firstly, the bulked segregating analysis was used for linkage analysis, where DNAs from 15 purple leaf and 15 green leaf individuals bulked into two DNA pools, and then those two DNA pools were screened with 300 polymorphic simple sequence repeats (SSRs), covering all the 12 rice chromosomes [18]. After finding candidate marker through bulk segregation analysis, primary mapping was performed, where 328 purple leaf F_2_ individuals were used and more *InDel* primers were designed to get the directions of the candidate region. After getting the directions of the candidate region, fine mapping was carried out to narrow down the candidate region by designing more *InDel* primers toward the candidate region using 442 individuals with purple leaf trait in the F_2_ population. Orthologous sequence differences between indica rice (*Oryza sativa* L.) and japonica rice (*Oryza sativa* L.) were exploited to design more polymorphic primers using DNASTAR Lasergene 14 (DNASTAR, Madinson, WI, USA) software and Primer premier 5.0 (PREMIER Biosoft, San Francisco, CA, USA) software. Functional annotations of the genes within the candidate region were explored from databases of RGAP (Rice Genome Annotation Project) [19]. The analysis of cis-acting elements in the promoter was discovered by following the ‘PLACE’ database [20]. The primers used for the gene mapping are listed in Table S1.

### 2.3. Cross Section and Cellular Study

The leaf sections of fresh leaf blade were cut by using a common razor blade and fixed in Formalin Aceto Alcohol (FAA) 50% for 24 h at 4 °C and stored in 70% ethanol. After that, they were washed with 2% acetic acid and treated with dual coloration of Safranine and fast green and ultimately the prepared sections were observed under image PRO-PLUS v 6.0.

For transmission electron microscopy, fresh leaves at the tillering stage from both *pl6* and WT were cut using the blade and fixed in 2.5% glutaradehyde (Sangon Biotech, Shanghai, China) at 4 ℃ for 48 h. After washing the samples three times with phosphate buffer, they were treated with 1% (*w/v*) osmium tetroxide at 4 °C for 2 h and again washed three times with phosphate buffer. Then, the samples were dehydrated in a graded ethanol series (30%, 50%, 70%, 85%, 95%, 100% (*v/v*)) for 15 min at each concentration. The samples were placed in a 1:1 mixture of absolute acetone and the final Spurr resin mixture for 1 h at room temperature, then transferred into a 1:3 mixture of absolute acetone and the final resin mixture for 3 h and to final Spurr resin mixture for overnight. Samples were then placed in eppendorf contained Spurr resin and heated at 70 °C for more than 9 h. The samples were sectioned in LEICA EM UC7 ultratome and sections were stained by uranyl acetate and alkaline lead citrate for 5 to 10 min, respectively, and observed in Hitachi Model H-7650 TEM at Bio-ultrastructure analysis Laboratory of Agrobiology and environmental sciences, Zhejiang University.

### 2.4. Anthocyanin Contents and Total Soluble Sugar Measurement

The total anthocyanin contents of leaf blade at active tillering stages were quantified. Briefly, homogenous mixture 0.5 g of fresh tissues were prepared with extraction buffer and 1% HCl/Methanol (*v/v*), for 24 h at 4 °C with overnight shaking. The supernatant was taken after centrifugation at 10,000 rpm for 15 min at 4 °C and absorbance at 530 and 657 nm wavelengths were determined using a spectrophotometer UV-1800 (Shanghai, China). Total anthocyanin was quantified by using equation: Q_Anthocyanins_ = (A530 − 0.25 × A657) × FW^−1^. Total soluble sugar (TSS) extraction was carried out with 80% ethanol from randomly five plants and quantified by taking supernatants [21]. Measurements were repeated three times as replications.

### 2.5. Determination of Antioxidant Activity and Total Antioxidant Compounds

The total phenolic content was measured by the Folin Ciocalteu method using gallic acid as a reference compound [22]. The content of total phenolic compounds was expressed as mg of gallic acid equivalents per g of fresh weight (mg GAE. G^−1^ FW). The flavonoids content was determined by the aluminum trichloride method using catechin as reference compound [23]. The total flavonoids content was expressed as (g E catechin.100 g^−1^ FW). All the experiments were repeated three times. The mean values and standard deviations were calculated using the Microsoft Excel software (Microsoft Corporation, Redmond, WA, USA).

### 2.6. Chlorophyll Content, Chlorophyll Flouresence and Photosynthetic Parameters

The chlorophyll a(Chl a), chlorophyll b (Chl b) and total chlorophyll contents of both *pl6* and WT were measured from flag leaves at tillering, heading and grain filling stages using the Arnon method [24]. Chlorophyll fluorescence was measured by an imaging pulse amplitude modulated fluorimeter (IMAG-MAXI; HenizWalZ, Effecl-trich, Germany) after keeping the leaves in dark for 30 min [25]. The maximal quantum efficiency of Photo-system II (PSII; Fv/Fm) for *pl6* and WT was measured using three times repetitions. In addition, photosynthetic rate (Pn), stomatal conductance (Gs), transpiration rate (Tr), and intracellular CO2 concentration (Ci) were measured from 10 to 11 am at the tillering stage using the LI-6400 portable photosynthesis system (LI−COR, Lincoln, NE, USA). 

### 2.7. Measurement of Endogenous Phytohormones

Endogenous gibberellic acid (GA), abscisic acid (ABA), cytokanin (CK) and salicylic acid (SA) were measured by using a plant ELISA kit (Shanghai Enzyme-linked Biotechnology Co. Ltd., Shanghai, China) following the producer’s instructions. In order to measure the concentration of GA, ABA, CK and SA in the samples, *pl6* and WT leaves were ground in liquid nitrogen. The GA, ABA, CK and SA ELISA kit contains a set of calibration standards. The calibration standards and samples assayed at the same time, whereas the standard curve was sketched using the concentration of calibration standards against optical density (O.D.). The concentrations of GA, ABA, CK and SA in the samples were then established by tracking standard curve and setting the similar trend in samples.

### 2.8. RNA Extraction and Quantitative qRT-PCR Analysis

Total RNA was extracted using Trizol reagent following the manufacturer’s protocol (Invitrogen, Carlsbad, CA, USA). The instructions of the Prime Script Reverse Transcript reagent Kit (Takara, Japan) were followed to synthesize cDNA with lateral use of gDNA Eraser and SYBR Premix Ex *Taq* II before performing qRT-PCR (Quantitative Real Time-PCR). Primers for qRT-PCR are listed in the Table S2. The reaction solution (10 μL) contained 1 μL of both forward and reverse primers (10 μM), 5 μL SYBR Premix Ex *Taq* II, 1 μL cDNA and 2 μL ddH_2_O. PCR was performed in three steps: activation at 95 C for 30 s, followed by 40 cycles of denaturation at 95 C for 5 s, annealing at 55 C for 20 s, and extending at 72C for 10 s. The detection of amplification and data were processed through Real-Time System of Roche Light Cycler ^®^ 96 (Basel, Switzerland) and the relative expression was calculated by 2^−ΔΔCT^ method after normalization of cq values of genes using the cq values of the rice actin gene [26]. Values of expression levels represent the means standard deviation (SD) of three biological replicates. * *p* < 0.05.

### 2.9. Statistical Analysis

All data are shown as means standard deviation (SD) of at least three replicates. The statistical software package SPSS (version 20, IBM corporation, Armonk, North Castle, NY, USA) was used for data analysis. One-way analysis of variance and subsequently a Tukey’s test were performed for pair-wise statistical significance difference. The graphs were prepared using Origin Pro (version 8.0, Origin Lab Corporation, Wellesley Hills, MA, USA). 

## 3. Results

### 3.1. Phenotypic Characterization of pl6 Mutant

**Figure 1:**
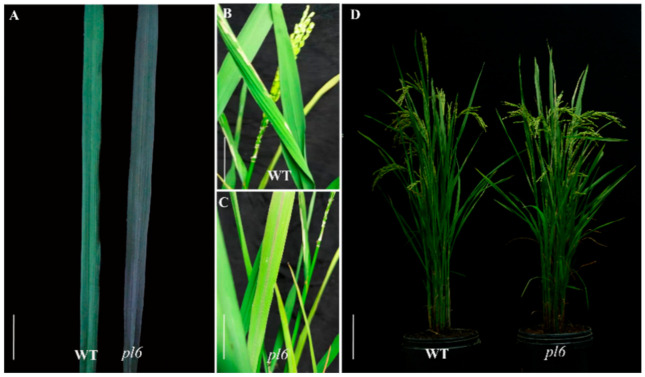
Morphological features of the wild type (WT) and *pl6* mutant at different growth stages. (**A**) The leaf at the tillering stage, bar = 2 cm; (**B**,**C**) The leaves of WT and *pl6* mutant at late Heading stage, respectively; bar = 5 cm; (**D**) The plant at late grain filling stage, Bar = 10 cm.

The *pl6* mutant phenotype was recognized visually by perceiving the purple color in the leaf blade of the mutant at M_2_ generation. The leaves in the *pl6* mutant were green at the seedling stage, gradually turned to purple at the early tillering stage, stayed purple to the late tillering stage, and vanished from the leaf blade at the early heading stage (Figure 1A). Meanwhile, the purple color lingered in the purple midrib of *pl6* up to the late heading stage, and disappeared at the grain filling stage (Figure 1B,C). 

### 3.2. Genetic Analysis and Map-Based Cloning of OsPL6 Gene

All F_1_ plants from the cross of *pl6* and its WT uniformly exhibited a green color in the leaf blade, indicating recessive inheritance of the trait. Among 423 F_2_ individuals, 120 individuals were purple, and 303 individuals showed similarity with WT. The segregation in the F_2_ population showed a good fit to the 3:1 ratio (χ^2^ = 2.52 < χ^2^_0.05_ = 3.84), suggesting that the purple leaf trait in *pl6* was controlled by a single recessive nuclear gene.

**Figure 2:**
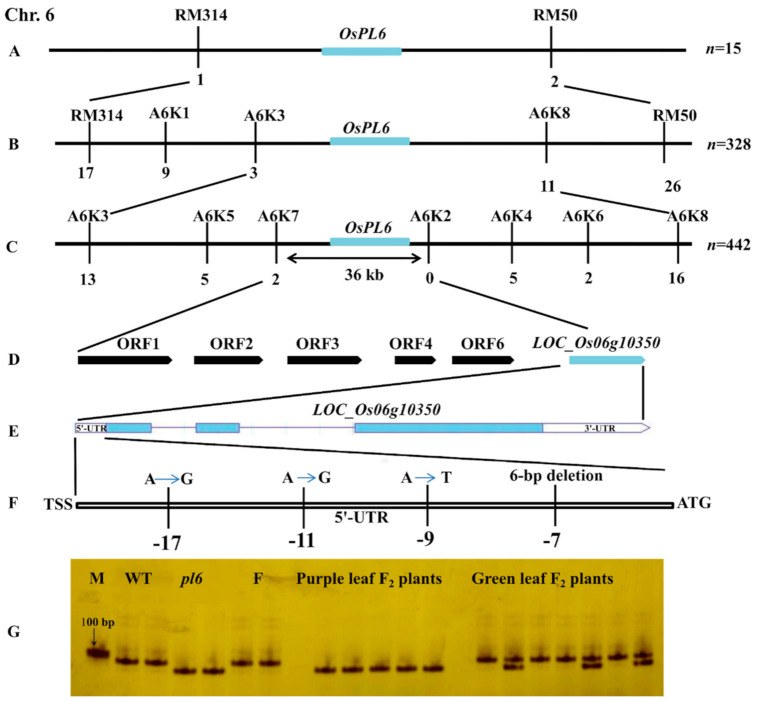
Map-based cloning of the *OsPL6* gene. (**A**) The *OsPL6* gene was located between markers RM314 and RM50 on chromosome 6 by Bulked segregate analysis (BSA); (**B**) Primary map of *OsPL6* graphed to a section between the InDel markers of A6K3 and A6K8 by using 328 F_2_ individuals of *pl6*; (**C**) Fine mapping of *OsPL6* to a gap of 36 kb between the markers A6K7 and A6K2 through 442 F_2_ individuals of *pl6*; (**D**) Six putative genes were predicted in the 36 kb section, and *LOC_OsO6g10350* was hypothetical to be *OsPL6*; (**E**) The structure of the *OsPL6* gene where the boxes implied exons and lines pointed as the introns; (**F**) Three substitution and one 6-bp deletion were found on 5′-UTR region of the gene; (**G**) The detection of sequence polymorphism with indel marker. M, marker (DL2000 marker, Takara Biomedical Technology, Beijing, China); *pl6*, mutant; F, father (Zhenong 104); WT, wild type (Zhenong 41).

During the map-based cloning, a total of 795 F_2_ individuals, obtained from the cross of *pl6* and Zhenong 104, were used for the mapping. Firstly, the bulked segregation analysis (BSA) was carried out using SSR and InDel markers on the 12 rice chromosomes. We identified the candidate region between two SSR markers RM314 and RM50 on the short arm of chromosome 6 based on the polymorphic marker between the parents and the F_2_ population (Figure 2A and Table S1). Furthermore, 328 individuals with purple leaves were used to primarily map the target gene between two designed InDel primers A6K3 and A6K8 (Figure 2B). For further fine mapping, 442 individuals with purple leaves were used to trap the target gene by narrowing down the distance between A6K3 and A6K8. Finally, it was confined up to the 36 kb region between InDel markers A6K7 and A6K2 (Figure 2C). The mapped region contained six putative genes, encoding F-box domain containing protein (*LOC_Os06g10290*), LRR containing protein (*LOC_Os06g10300*), growth regulating factor protein (*LOC_Os06g10310*), myb-related protein (*LOC_Os06g10330*), autophagy-related protein (*LOC_Os06g10340*) and MYB family transcription factor (*LOC_Os06g10350*)*,* respectively (Figure 2D and Table S3). To examine the gene responsible for purple leaf blade, we sequenced and blasted all 6 genes from the 1 kb upstream to downstream of each gene between the *pl6* and WT. Interestingly, there was no diversity for five genes except *LOC_Os06g10350*, encoding MYB transcriptional activator [27], which showed the mutations in the 5′-UTR region (Figure 2F). For further confirmation of the target gene, we designed a marker covering the 5′-UTR region of *LOC_Os06g10350*. The band size of marker was smaller in the *pl6* mutant as compared to WT (Figure 2G). In the F_2_ population derived from *pl6*/Zhenongda 104, we observed that the individuals with purple leaves had same band size with *pl6* mutant, while the individuals with green leaves presented the same band size as WT or the heterozygous samples (Figure 2G). Furthermore, we could not find any individual with green leaves showing the same band size with *pl6* mutant. With the combination of the mapping, sequencing and further marker confirmation, we regarded *LOC_Os06g10350* as the target gene and named it *OsPL6*. Moreover, the sequence results showed that there are also many mutations in the promoter region; however, sequence blast on the PLACE database revealed that most of the mutation remains silent without adding or deleting any cis elements in the promoter. Yet, four new cis-elements were inducted in the promoter region and one cis-element was lost from the 5′-UTR region of *pl6* (Figure S1). The single nucleotide polymorphism (SNP) at −702 bp caused the induction of cis-element CAAT box by mutating the sequence from CTAT of WT to CAAT in *pl6*. RBCSCONSENSUS cis-element inducted at −598 bp by sequence conversion from AATCTAA to AATCCAA in *pl6*. Introduction of SEF4MOTIFGM7S cis-element took place at −450 bp and sequence mutated from AATTTTG to ATTTTTG. Insertion of single base pair mutated GTGGTAG to GTGGTTAG and inserted SV40COREENHAN cis element at −119 bp of *pl6*. Three SNPs and one deletion altered the sequence from GAGAGAGAGAGAGAGA to GAGAGGGAGAGGGTG at −22 in 5′-UTR region ultimately lost GAGA8HVBKN3 cis element from *pl6*. These results suggest that the addition of new cis elements in the promoter could be responsible to activate the *OsPL6* gene to regulate anthocyanin in leaves. 

### 3.3. OsPL6 Mutation Regulate the Expression of Anthocyanin Biosynthesis Pathway Gene 

**Figure 3:**
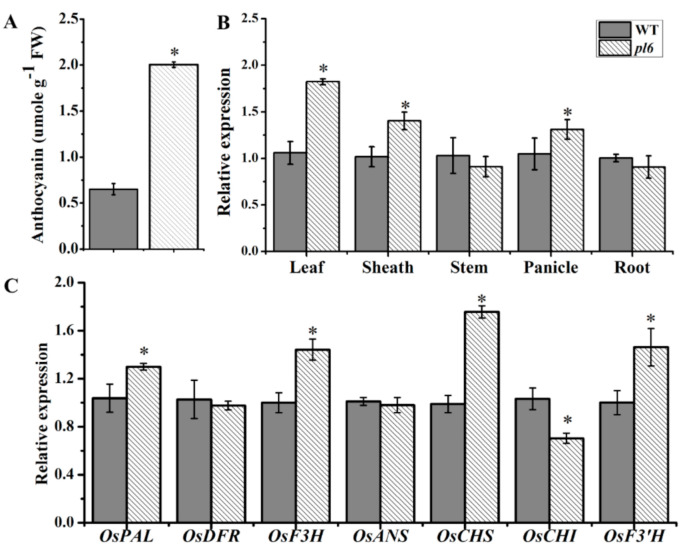
Estimation of anthocyanin contents, tissue-specific expression of *OsPL6* gene and related gene expression at the tillering stage. (**A**) Anthocyanin contents in leaves of WT and *pl6*; (**B**) The expression pattern of *OsPL6* gene in different organs; (**C**) The expression profile of anthocyanin biosynthesis pathway genes between WT and *pl6* at grain filling stage. * *p* < 0.05 following Tukey’s test. All data represent the mean ± SD of three replicates.

Anthocyanin contents were measured at the tillering stage because the leaf coloration started to attain at this stage. The anthocyanin contents in the leaf blade of the *pl6* mutant accumulated significantly higher than that in WT (Figure 3A). The expression profile of *OsPL6* carried out in different tissue by qRT-PCR. The results show that *OsPL6* expression was significantly increases in leaf, sheath and panicle of *pl6* as compared to WT (Figure 3B). 

To find out the influence of *OsPL6* as an MYB transcriptional activator on structural genes in the anthocyanin biosynthesis pathway, the transcript levels of *OsPAL, OsCHS, OsCHI, OsF3H, OsDFR, OsANS2* and *OsF3′H* in the WT and *pl6* were measured by qRT-PCR. The anthocyanin biosynthetic genes, *OsPAL*, *OsCHS*, *OsF3H* and *OsF3′H* were up regulated in the *pl6* mutant compared with WT, whereas *OsCHI* was down-regulated in the *pl6* mutant (Figure 3C). Reasonably, *OsPL6*, the regulatory gene, showed a significantly higher transcription level in *pl6* compared to WT, indicating that anthocyanin biosynthetic genes, *OsPAL, OsCHS, OsF3H* and *OsF3′H* were up-regulated by the activation of Myb transcription factor gene, guided to the accumulation of anthocyanin in the leaf blade of *pl6* mutant.

### 3.4. Anthocyanin Accumulation Altered Leaf Thickness and Chloroplast Ultrastructure

**Figure 4:**
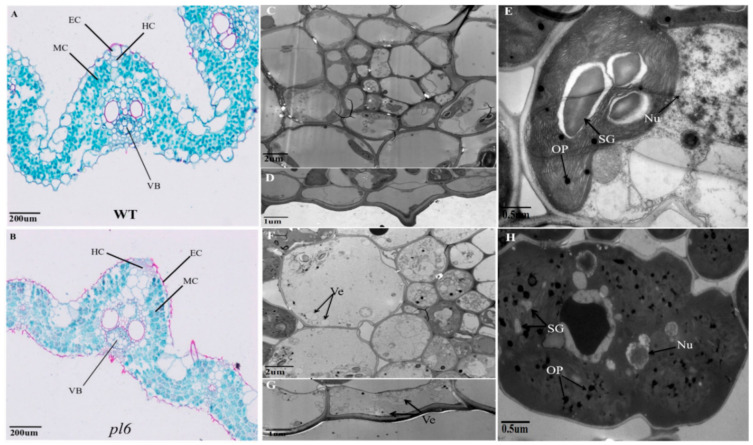
Transverse section and transmission electron microscopic images of the leaf blade between the WT and the *pl6* mutant; (**A**,**B**) Transverse section of a leaf blade of WT and *pl6* at the late tillering stage; (**C**,**F**) Transmission electron microscopic images of vascular cells at the late tillering stage, respectively; Bar = 2 μm; (**D**,**G**) Closed up images of epidermal cells, Bar = 1 μm; (**E**,**H**) Ultrastructure of mesophyll cells in the leaf of WT and *pl6*, Bar = 0.5 μm; MC, Mesophyll Cells; HC, Hypodermis cells; EC, Epidermal cells; VB, Vascular bundles; SG, Starch granules; OP, Osmophilic Plastoglobuli; Nu, Nucleous; Ve, Vesicles, respectively.

The transverse section of leaf blade showed that anthocyanin accumulation in leaf blade of *pl6* increase the leaf thickness and created an observable difference between WT and *pl6*. The number of cells in hypodermis, epidermis and mesophyll vascular bundles were increased in the *p16* mutant, whereas the cell area remained the same. The results indicate that the greater cell number could be responsible for proliferating leaf thickness in the *pl6* mutant (Figure 4A,B). The transmission electron microscope (TEM) was used to observe epidermal cells, vascular cells and mesophyll cells of the leaf blade of WT and *pl6* (Figure 4C,D,F,G). The results show that epidermal cells and vascular cells of *pl6* have a lot of vesicles which could not be observed in WT. In addition, the results show a lot of changes in ultrastructure of mesophyll cells. The chloroplast in *pl6* was merged with cytoplasm and seemed to have no membrane around it, whereas chloroplast was clearly separate from cytoplasm in WT (Figure 4E,H), indicating that chloroplast development was deteriorated in *pl6*. The starch granule in *pl6* was also not clearly visible in chloroplast and looks smaller in size as compared to the WT. The plastoglobuli of WT was normal in size and round in shape, but its size and shape was irregular in *pl6*, suggesting that chlorophyll metabolism was affected in the *pl6* mutant. The nucleus in the cytoplasm of *pl6* was also hidden and difficult to observe, but it was obvious in WT. In addition to the nucleus, other organelles in the cytoplasm were also not clearly visible in the *pl6* mutant.

### 3.5. OsPL6 Activation Reduce the Chlorophyll Contents and Related Genes Expression 

**Figure 5:**
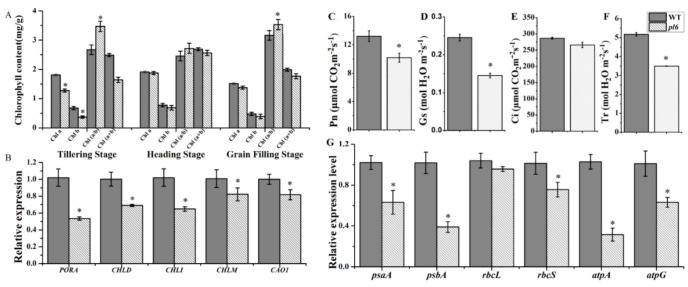
Photosynthetic pigment contents, photosynthetic parameters and the expression of relative genes between the WT and the *pl6* mutant. (**A**) Pigment contents in leaves at the tillering and heading, grain filling stage; (**B**) The expression prototype of pigment metabolism genes at the late tillering stage; (**C**) Photosynthetic rate (Pn); (**D**) Stomatal conductance (Gs); (**E**) Intercellular CO_2_ concentration (Ci) and (**F**) transpiration rate (Tr); (**G**) Expression analysis of genes associated with photosynthesis. Asterisks indicate the statistical significance levels according to Tukey’s test: * *p* < 0.05. All values represent the mean ± SD of three replicates.

To determine the effect of higher anthocyanin accumulation on pigment metabolism in purple leaf mutant, chlorophyll contents were measured at different growth stages in WT and *pl6.* Chlorophyll a, chlorophyll b, and total chlorophyll contents were significantly reduced at the tillering stage, when a purple color appeared in the leaf blade. It became normal at the heading stage, when the purple color almost disappeared (Figure 5A). Chlorophyll contents were relatively low at grain filling stage as compared to first two stages, indicating slight senescence in the *pl6* mutant. 

Subsequently, the expression analyses of pigment metabolism-related genes were compared between *pl6* and WT plants at the tillering stage (Figure 5B). The transcription levels of chlorophyll metabolism-related genes *PORA* (encoding NADPH-dependent protochlorophyllide oxidoreductase), *CHLD* (encoding Mg-chelatase D subunit), *CHLI* (encoding Mg-chelatase I subunit), *CHLM* (encoding Mg-protoporphyrinmethyl transferase) and *CAO1* (encoding chlorophyllide an oxygenase) were significantly down-regulated in *pl6*. The results suggest that *OsPL6* affected chlorophyll metabolism in the *pl6* mutant. 

### 3.6. Light Attenuation Feature of Anthocyanin Reduces the Photosynthetic Efficiency 

The photosynthetic characteristics in flag leaves were observed by gas-exchange measurement *pl6* and WT (Figure 5C,F). Photosynthetic rate (Pn), stomatal conductance (Gs), and transpiration rate (Tr) in *pl6* were significantly decreased, while intercellular CO_2_ concentration (Ci) remained unchanged as compared to WT.

**Figure 6:**
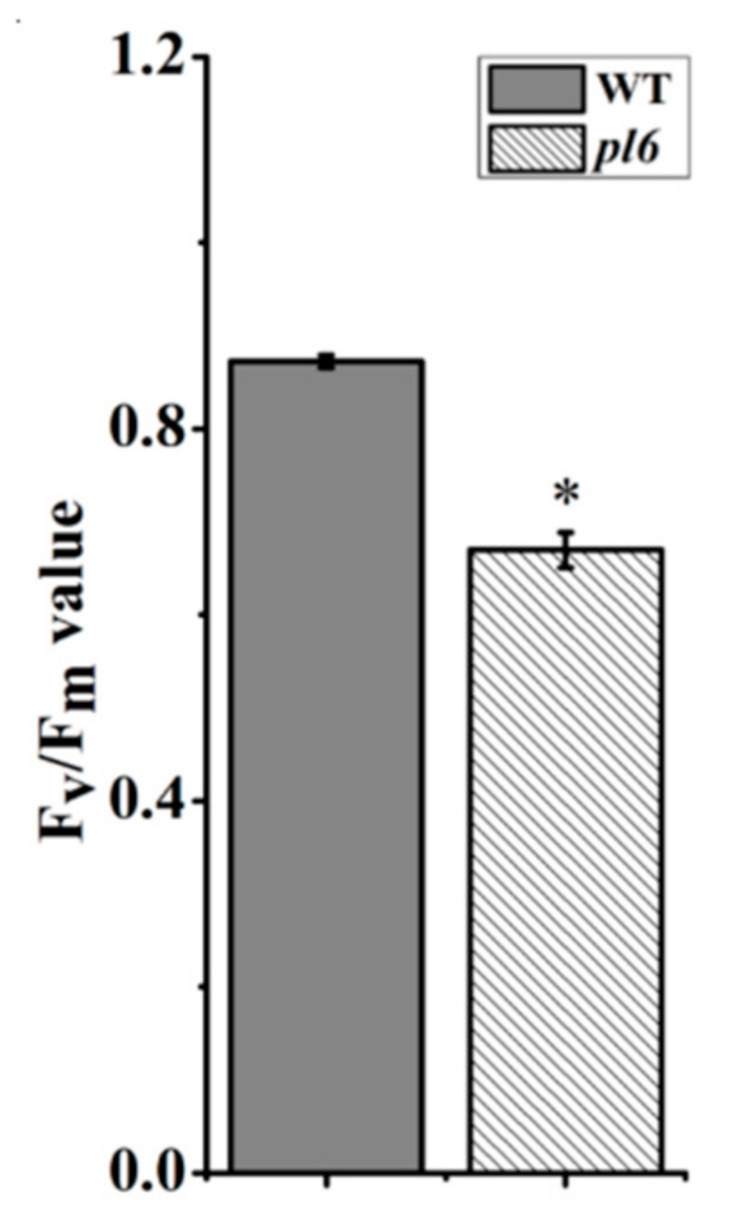
Detection of chlorophyll flouresence between the WT and the *pl6* mutant at the tillering stage. All values are the mean ± SD of three biological replications. * *p* < 0.05 by Tukey’s test.

Chloroplast development and photosynthesis-related genes were also compared between *pl6* and WT plants (Figure 5G). The transcription levels of *rbcS* (encoding the small sub-unit of Rubisco), *psaA* (encoding the reaction center protein A1 of photosystem I (PSI)), psbA (encoding the reaction center protein D1 of PSII), *atpA* (encoding the α subunit of ATP synthase), *atpG* (encoding the γ subunit of ATP synthase) were significantly down-regulated, while *rbcL* (encoding the large subunit of Rubisco) were not affected. The results indicate that *OsPL6* influenced the chloroplast development and photosynthesis badly in *pl6* mutant plants. For testing chlorophyll fluorescence, Fv/Fm (the maximal competence yield of PSII) was measured. The Fv/Fm was reduced in the *pl6* mutant as compared to the WT, suggesting abnormal chlorophyll metabolism (Figure 6).

### 3.7. Photoabatement Mechanism of Anthocyanin Enhance the Antioxidant Activity of Leaves 

**Figure 7:**
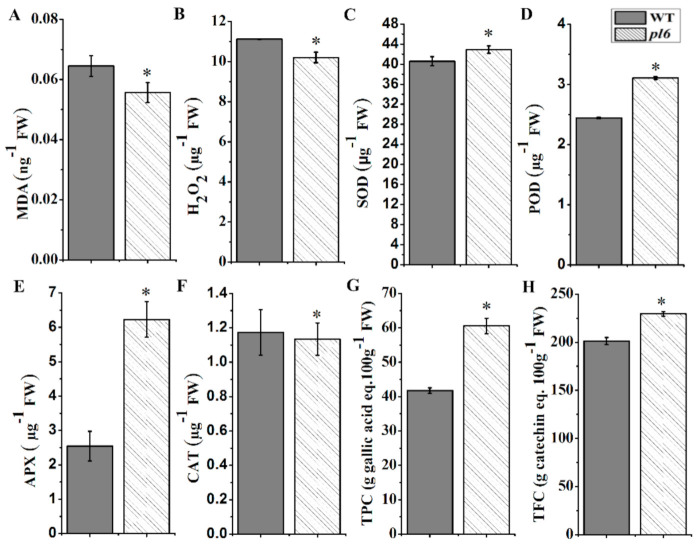
Antioxidant activity and oxidative damage between the WT and the *pl6* mutant. (**A**) SOD (superoxide dismutase); (**B**) APX (ascorbate peroxidase); (**C**) POD (peroxidase); (**D**) CAT (catalase); (**E**) TFC (total flavonoid contents); (**F**) TPC (total phenolics content); (**G**) H_2_O_2_ hydrogen per oxide; (**H**) The MDA (malonaldehyde) contents. All values are mean ± SD of three biological repeats. * *p* < 0.05 followed by Tukey’s test.

In order to investigate the antioxidant activity of purple leaves as compared to the green leaves in WT, we measured reactive oxygen species (ROS) including H_2_O_2_, MDA and their scavenging antioxidants including enzymatic antioxidants and low molecular weight antioxidants (Figure 7A,B). The results showed that H_2_O_2_ and MDA contents were significantly less in purple leaf as compared to green leaf in WT, indicating lower oxidation damage in purple leaf. Oxidative species could be scavenged by enzymatic antioxidants such as superoxide dismutase (SOD), peroxidase (POD), and ascorbate peroxidase (APX) were significantly higher in purple leaf while CAT is significantly higher in green leaves (Figure 7C–F). The purple leaves also held a higher amount of non-enzymatic antioxidants, including anthocyanin, total phenolics compounds and total flavonoids (Figure 7G,H). These results suggest that *pl6* mutant was better equipped with antioxidant activity as compared to the WT.

### 3.8. Anthocyanin Accumulation Improved the Hormonal Signaling

**Figure 8:**
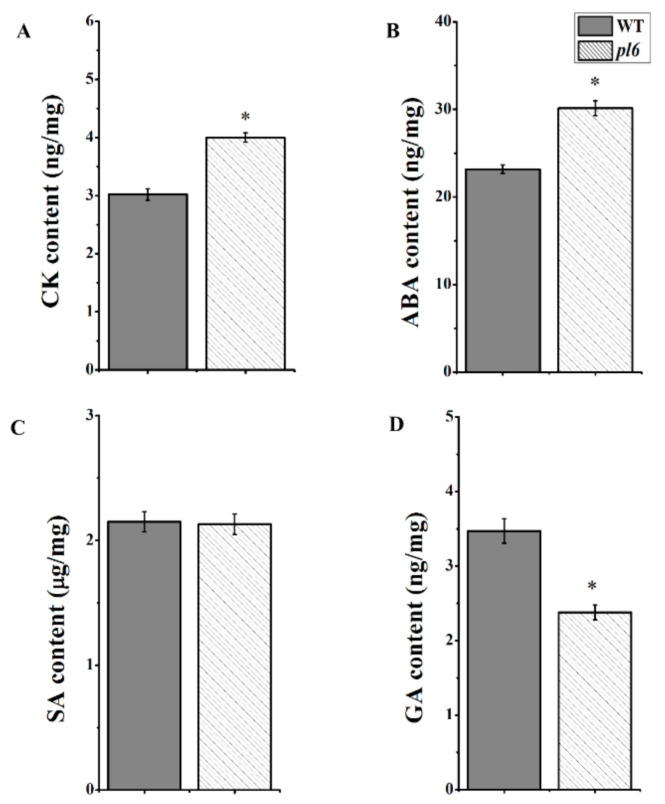
Detection of various phytohormones of the WT and the *pl6* mutant. Cytokanin (CK) (**A**), abscisic acid (ABA) (**B**), salicylic acid (SA) (**C**) and gibberellic acid (GA) (**D**) content of the top of flag leaves at the late tillering stage. All values are the mean ± SD of three biological replications. * *p* < 0.05 by Tukey’s test.

**Figure 9:**
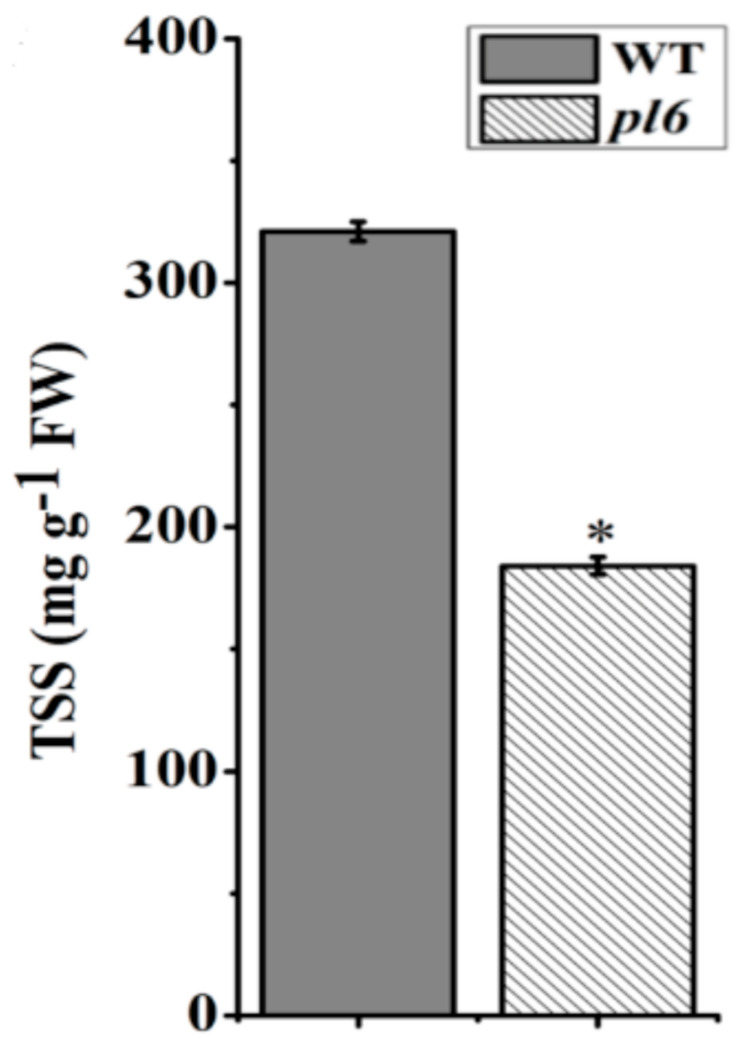
Detection of total soluble sugar (TSS) contents between the WT and the *pl6* mutant at the late tillering stage. All values are the mean ± SD of three biological replications. * *p* < 0.05 by Tukey’s test.

The levels of the most common growth-related phytohormones, including GA, ABA, CK, and SA in the plant were measured from the rice leaf blade of the *pl6* mutant and WT. The results showed that GA levels were significantly decreased in the *pl6* mutant and levels of CK and ABA contents were significantly elevated, but there was no significant change in SA compared with WT (Figure 8A–D). The results suggest that the *pl6* mutant had better hormonal signaling for anthocyanin accumulation. In addition, total soluble sugar (TSS) content reduced in the *pl6* mutant compared with the WT (Figure 9), which was contradicted to sugar-hormone cross talk in anthocyanin biosynthesis [28].

## 4. Discussion

Purple leaf color is an important morphological marker and valued as an important trait for the study of rice domestication and breeding [29]. A growing body of experimental evidences suggest that anthocyanin certainly equip the plants with physiological benefits. However, the mechanism has not been fully exploited. Anthocyanin biosynthesis is regulated by structural genes, yet their expression intensity is controlled by interaction of regulatory genes, which were encoded by transcription factors (TFs) known as MBW complex [9]. Mutation in any of the TF of this complex can enhance or suppress the expression of structural gene. For instance, a single base pair insertion in Myb TF activate the anthocyanin biosynthesis in leaf blade [30]. Leaf color mutants are valuable resources for further isolation and characterization of regulatory genes. Here, we attempted to develop purple leaf mutant *pl6* with treatment of gamma rays and phenotypic difference were observed in leaf color at the tillering stage. Using a bulk segregation analysis and map-based cloning; we fine mapped the candidate gene within the 36 kb region on the short arm of chromosome 6. The *OsPL6* gene, encoding Myb TF, was identified as candidate gene, responsible for purple coloration in the leaf blade of rice. Our sequencing results indicate the addition of four new cis acting elements and the loss of one element caused by mutation in promoter region of *OsPL6*. The contribution of inducted CAAT box, SEF4MOTIFGM7S, SV40COREENHAN and GAGA8HVBKN3 cis acting elements in the *pl6* mutant might be consistent with their influence reported in promoters of different species in past [31,32,33,34]. Induction of these four cis elements had not only compensate the loss of GAGA8HVBKN3 activity but also increase the mRNA transcript level of *OsPL6* and associated structural genes to accumulate anthocyanin in leaves.

Our results from both chlorophyll contents and transmission electron microscopy reveal that anthocyanin had negative impact on photosynthetic machinery of purple leaf compared with green leaves of wild type (Figure 5A or Figure 4H). Purple leaves exhibit more anthocyanin accumulation (Figure 3A), which could absorb up to 17% of green light [35]. The previous study implied that green light derives photosynthesis more efficiently as compared to other lights of solar radiation [36]. Light attenuation by anthocyanin could attribute to degradation of thylakoid membrane resulted in higher number of plastoglobuli (Figure 4H). Similarly, [37] associated the higher number of plastoglobuli with the disintegration of grana and stroma thylakoids. Moreover, the reduction in chlorophyll content and chlorophyll fluorescence might be caused by the disintegration of thylakoid attributed due to less availability of green light shielded by anthocyanin. Similar effects of anthocyanin for light attenuation was noted for purple pods of *Bauhinia variegate* [38]. Furthermore, many researchers in the past have observed a decline in photosynthetic efficiency linked with anthocyanin accumulation, although they could not provide the clear mechanism. Consistent with their findings, our results show poorer photosynthetic activity for purple leaf (Figure 5C,F), which could be due to decreased chlorophyll contents [39]. Additionally, the fall of transcript level of genes associated with pigment metabolism and photosynthesis showed the diminishing effects of anthocyanin at the gene level to interrupt photosynthetic machinery (Figure 5B,G). Previously, the similar result was observed that anthocyanin accumulation down-regulated the genes allied with photosynthesis [30]. Thus, anthocyanin contributed to disturb the photosynthesis efficiency, thereby reducing the chlorophyll contents.

Anthocyanin accumulation in the *pl6* mutant decreased the levels of MDA and H_2_O_2_ as compared to the WT which portrayed that purple leaves are better equipped with antioxidant activity (Figure 7A,B). Earlier study suggested that photo abatement mechanism of anthocyanin have the ability to thwart the over-excitation of the electron transport chain yet diminish the oxide generation [40]. Besides, the previous study showed anthocyanin as a strong antioxidant [41]. Accordingly, our results show the higher value of anthocyanin in the *pl6* mutant that could detoxify the harmful oxides with the impact of putative mechanism of light attenuation and ROS scavenging. Moreover, it has been reported that oxides generated by photoreduction can be disproportionate to H_2_O_2_ via reaction catalyzed by the SOD family [42], which could further scavenged by APXs to ascorbate it into water [43]. Consistent with these findings, our results show a higher amount of SOD activity for purple leaf (Figure 7C), which is coupled by enhanced APX contents to detoxify the production of H_2_O_2_ (Figure 7D). It has also been noted that more production of H_2_O_2_ can trigger signal for anthocyanin biosynthesis to enhance tolerance to stress [44]. In our case, lower H_2_O_2_ content attributed the anthocyanin accumulation purely to high transcript level of *OsPL6* gene. Our microscopic results exposed that the nucleus was not clear enough in mutant (Figure 4H), that could be due to the covering of nucleus by anthocyanin to protect DNA from oxidative stress and harmful radiations [45]. Hence, anthocyanin can be considered as effective antioxidant to protect the plant from oxidative damage.

Several lines of experimental evidence suggested that the positive relation of anthocyanin with ABA hormone [46,47]. Shen et al. suggested that Myb played an important role in ABA-regulated anthocyanin biosynthesis, and ABA was a signal molecule that promoted red-colored sweet cherry fruit by accumulating anthocyanin [48]. Similarly, our results show that higher transcript level of Myb and increased level of ABA, which could regulate anthocyanin accumulation in the *pl6* mutant (Figure 8B). Moreover, our results also indicate an increased level of CK for purple leaves, which could also affect anthocyanin accumulation (Figure 8A), consistent with the research that that Myb transcription factors influenced the cytokinin-regulated anthocyanin biosynthesis [49]. However, unlike other hormones, GA showed a reduction due to the anthocyanin synthesis in the *pl6* mutant (Figure 8D), substantiating the hypothesis of negative association of GA with anthocyanin [50]. Thus, the higher transcript level of Myb regulated the hormone-regulated anthocyanin biosynthesis.

## 5. Conclusions

A rice mutant, *pl6*, was characterized by its phenotype consisting of purple leaves with a purple midrib at the onset of heading. The mutant had degraded chloroplasts, and reduced chlorophyll contents. A genetic analysis confirmed that the *pl6* trait is controlled by a single recessive nuclear gene, the result of mutations in promoter region of *OsPL6*. This gene was fine-mapped to a 36 kb interval between the short arm of chromosome 6 and further confirmed by marker development. The higher transcript level of the Myb gene (OsPL6) as well as the increased contents of ABA and CK contents in *pl6* could be responsible for anthocyanin biosynthesis. As this gene has not been reported for regulating anthocyanin accumulation in leaves before, this study thus might provide a morphogenesis and molecular basis for elucidating the role of *OsPL6* in controlling the purple color in leaves. These findings deepen our understanding of the mechanisms of anthocyanin accumulation in leaves and their antioxidative role.

## Supplementary Materials

The following are available online at https://www.mdpi.com/2223-7747/9/11/1477/s1, Table S1: List of the primers used in this study for gene mapping and sequencing, Table S2: List of the primers used in this study for qRT PCR, Table S3: The genes information between 36 kb target region, Figure S1: Sequence analysis of the promoter and5’ UTR regions of WT and pl6in PLACE database.
