# Supplementary material for: The Purple Leaf (pl6) Mutation Regulates Leaf Color by Altering the Anthocyanin and Chlorophyll Contents in Rice
Source: Plants (Basel). 2020 Nov 3;9(11):1477. doi: 10.3390/plants9111477 (PMC7693866; doi:10.3390/plants9111477)
Supplement: Supplementary file 1 [file plants-09-01477-s001.pdf]

Supplementary Tables

Table S1. List of the primers used in this study for gene mapping and sequencing.

Marker	Forward primer (5′→3′)	Reverse primer (5′→3′)	Use
RM314	CTAGCAGGAACTCCTTTCAGG	AACATTCCACACACACACGC	Mapping
RM50	ACTGTACCGGTCGAAGACG	AAATTCCACGTCAGCCTCC	Mapping
A6K1	TTTCCCTCGTTTGATGACTT	GCCCCAATAATAATGGTAAG	Mapping
A6K2	GCTCTGCGACTGATGAACT	AACGTGACAAACCTATTGCT	Mapping
A6K3	ACACATCCGTCGCACTCA	GGAGCCAAGAAGACGTGAAG	Mapping
A6K4	TCTCGGTCTCATGGTCACC	TTTGGGAGGATTGGGAAT	Mapping
A6K5	CATCCAAATAGGTTGCGAA	GTGGGACCCACATGTCAT	Mapping
A6K6	AGTGCGGCAATTATTGAT	CTGAAGTCTGAAGACCGAAT	Mapping
A6K7	CATGACAACAATGGGTATGAC	CAGATATTATGAGGAATCAG	Mapping
A6K8	GTTGTCAGGTGGTCTCTCATT	CCGTTGCTGTGTCGGTGT	Mapping
PL1	TCAGTTTCAGAGAGAGGAGTC	TCATTCTTGGACTAGAGTTCA	Sequencing
PL2	TTATCCCTTCAATACCCTTTAC	AAGAACATTGCATATCAGGC	Sequencing
PLM	CTCAGTCTCACACCGCACA	CAAGCTCTCCTCCCCATT	Marker

Table S2. List of the primers used in this study for qRT PCR.

Name	Genebank accession	Forward primer (5′–3′)	Reverse primer (5′–3′)
Actin	XM_015774830	GTGGTCGCCCCTCCTGAAAG	GGCTTAGCATTCTTGGGTCCG
OsPL6	MK636605	CAACGAGCTGGTTTGAGGCGGT	TGTAGAGACCACCTGTTGCCGAG
OsPAL	XM_015769634	AGCTCCGTCAAGAACTGCGTC	CGATGGCGGTGAGGAGGT
OsANS	Y07955	AGCTGCTCGCCATCCTCTCC	GCTGACGTCGGTGTGTGCC
OsCHS	XM_015762568	GTTCACACCGCTGGGGATCT	TCCTCTCCTTGTCCAGCCCA
OsDFR	MK636607	GACATCGACTTCTGTCGCCG	TGACGCTGATGAGGTCCAGC
OsF3'H	XM_015757555	CTACAGTACCAGCCTTCTC	TCATCAGTGTGACCATCC
OsF3H	XM_015779149	TGACCATGCTCACTATCTG	TCTCTATCTTCCTTGTCTT
OsCHI	XM_034715967	CATCCTCTTCACCCACTC	GATGATGGAATCCAGCAC
CHLI	XM_015772495	AGTAACCTTGGTGCTGTG	AATCCATCAACATTCAACTCTG
CHD	XM_015775598	GCTTGCAGAAAGCTACACAAGC	AGGCCGTGAGCTAAAGGAGA
CHLM	XM_015785870	CCATCCATTGGTCTCCTTATGACA	GTAGCCTACTTACCATCAATGAGC
CAO1	XM_015758600	GACACCTTCATCTGGGCTTCAA	CGAGAGACATCCGGTAGAGC
PORA	XM_015759459	ATCACCAAGGGCTACGTCTC	GAGTTGTTGTTCCAGCTCCA
PSaA	AAS46121	TGGGGTTGATCCTAAGGAGATACCA	CCTCCGCGAAAACTAAGAAATTCTG
PSbA	AAS46104	GCGGTTCCCTATTCAGTGCTATG	TAACCATGAGCGGCCACAATATT
rbcL	AAS46127	GGAGGGACGTATGTCACCACAA	GAGTTACTCGGAATGCTGCCAAG
rbcS	X07515	GTGGCAACTAAGCCGTCATCGTC	TGCCTCACCCAACAACATATAGTC
atpA	AAP54723	TGAATCTCCTGCTCCGGGTATAA	TGCTGTTTTGCCGGTTTGTCT
atpG	AGT42322	AGGTGGAGCTCCTCTACTCCAAG	TCAGCTTCCCTTCCTTGGTGGT

Table S3. The genes information between 36 kb target region

Gene locus	Genebank accession	Putative function	Sequence alignment result
LOC_Os06g10290	BAS96685	F-box domain containing protein	No difference
LOC_Os06g10300	BAS96686	LRR containing protein	No difference
LOC_Os06g10310	BAS96688	Growth regulating factor protein	No difference
LOC_Os06g10330	N/A	Myb-related protein	No difference
LOC_Os06g10340	BAS96689	Autophagy-related protein	No difference
LOC_Os06g10350	BAF19004	MYB family transcription factor	Mutation in promoter region including SNPs at -612, -508 -360 and insertion at -29 as well as mutation in 5'-UTR region including 3 SNPs and 6-bp deletion

Supplementary Figures

Fig. S1. Sequence analysis of the promoter and 5' UTR regions of WT and *pl6* in PLACE database.

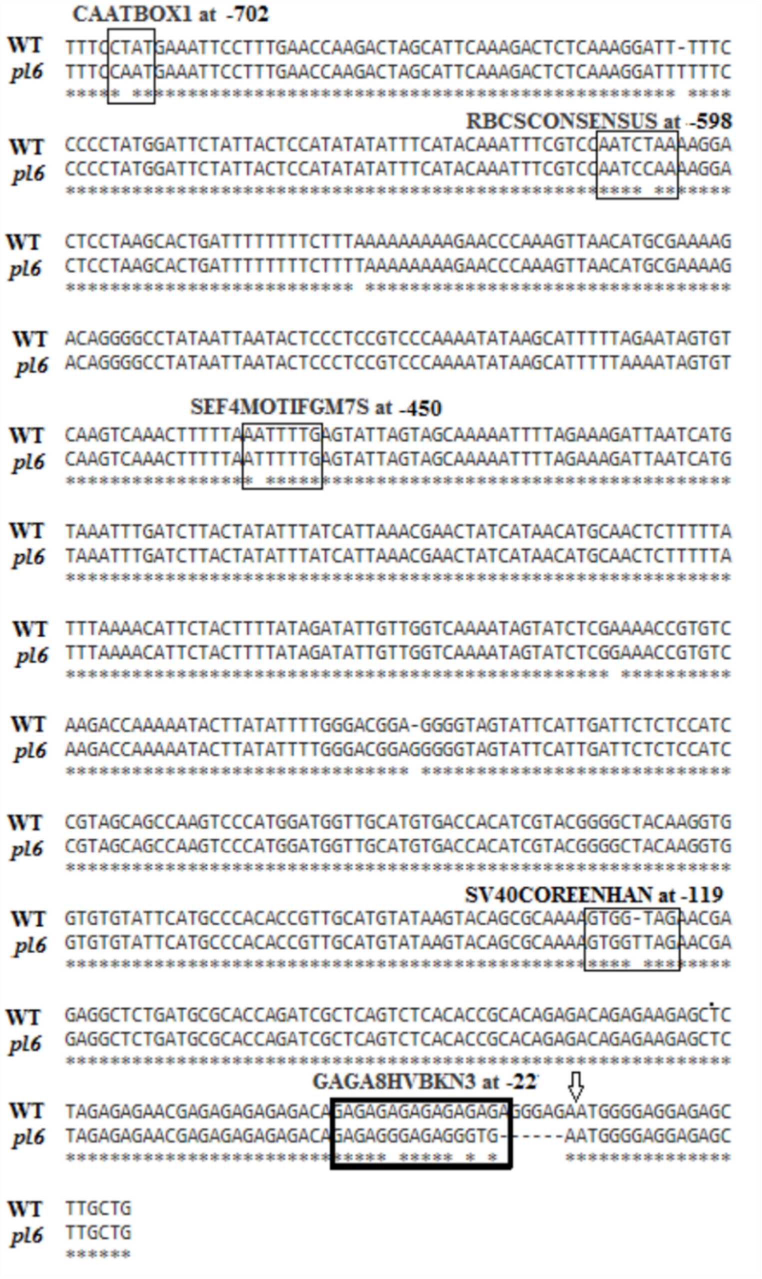

Normal black boxes show induction of new cis elements in *pl6* and bold black box show deletion of cis element from *pl6*. Black arrow showed start codon of gene.
